# The lived experience among Somali women of giving birth in Sweden: an interpretive phenomenological study

**DOI:** 10.1186/s12884-020-02933-9

**Published:** 2020-05-01

**Authors:** Susanne Wallmo, Karin Allgurin, Carina Berterö

**Affiliations:** 1 Women’s Health Care, Gynaecological Clinic Ryhov Region County Hospital, SE- 551 85 Jönköping, Sweden; 2 Maternity Ward, Gynaecological Clinic Ryhov County Region Hospital, SE- 551 85 Jönköping, Sweden; 3 Division of Nursing Science, Department of Medical and Health Sciences, Linköping University, SE- 581 83 Linköping, Sweden

**Keywords:** Pregnancy, Birth giving, Somali women, Interpretive phenomenological analysis, Confidence, Confirmed as a woman, Qualitative research

## Abstract

**Background:**

The health care-seeking behaviour among Somali women is different from Swedish women’s behaviour, and this may have consequences for birth giving. The aim of the study was to identify and describe Somali women’s lived experience of birth giving in Sweden.

**Methods:**

Qualitative individual interviews were conducted in Swedish with seven Somali women. The sample was purposeful, and the snowball sampling method was used. The interviews were digitally recorded and transcribed verbatim. Data were analysed using interpretative phenomenological analysis.

**Results:**

Four themes emerged during the analysis which revealed the Somali women’s lived experiences of giving birth in Sweden. a) Being recognised and confirmed as a woman. Somali women consider it important to be confirmed as a woman by the surrounding and professionals during pregnancy and birth giving. b) Communication is important for the women’s independence. There is a need to provide a structure for how this information is given and adaptation regarding content and format .c) Something naturally becomes unknown and complicated. Somali women come from a different culture, which affects their lived experiences of pregnancy and birth giving. There is a need for improved and clearer information for these Somali women regarding pregnancy and birth giving in another culture- the Swedish context d) Professional and competent taking care of. The women appreciate if they are treated with competency and professionalism; they do not want to be discriminated. The women feel confidence in health care when they meet competent and professional health care professionals.

**Conclusions:**

The findings in the study indicate that reproductive health care for Somali women should be improved with regard to cultural differences and lived experiences, as this affects their experience of pregnancy and childbirth in Sweden. There is a need for both knowledge and understanding in order to provide good quality care for these Somali women, especially those who have been genitally mutilated.

## Background

Global statistics on migration show that there were 244 million international migrants in 2015, which is about 3.3% of the global population. Out of these millions of international immigrants 48% were women [1]. In Sweden 2014, there were 19,424 Somali women in the age range of 16–49 years, and there were about 4074 in the age range of 25–29 years [2, 3]. Somali pregnant women in Sweden are a vulnerable group of patients, which shows that there are defects in the health monitoring system for foreign-born women. The health care seeking behaviour of Somali women differs from that of Swedish women and that may have consequences for the pregnancy [4]. There is knowledge about measured aspects of pregnancy and maternity care, but knowledge is sparse regarding the Somali women’s’ own experiences of birth giving in a Swedish context.

Many women from Somalia are not acquainted with prenatal and preventive care in their home country. They do not see the advantages of this kind of care and need to have big problems or become really sick to seek it [5]. The outcomes of pregnancy for women emigrating to Europe are much worse than for those women who are born or raised in that receiving country. The immigrated women have an essentially higher risk of giving birth to a child with low birth weight or giving birth prematurely. Women from Africa emigrating to Sweden are also at higher risk for perinatal death. This risk is higher when the women have been in Sweden for a short time and are first time mothers [6]. Somali women have an increased risk of prenatal death, longer deliveries, lower Apgar scores for the child, and more often finish their birth giving with a caesarean. This is because many Somali women have been genitally mutilated. Other reasons are culture differences, language and communication difficulties as well as differences in the concepts of health and illness [7].

There are some factors that are important for Somali women during pregnancy and birth giving; support from health care professionals (HCPs) and clear information. Information about giving birth is especially important. Those women who went through a Caesarean section felt powerless and had difficulties understanding why it was performed. Somali women also state that it is important to have information about alleviation of pain and its eventual side-effects [8]. However, statistics show that women from Somalia use less pain alleviation during birth giving than Swedish women; statistically significant figures regarding nitrous oxide (N_2_O) was used less often by the Somali women and epidural anesthesia for vaginal delivery was rarely used by the Somali women [4]. In Somalia, giving birth is a women’s issue only, something that is the women’s responsibility and which men have no part in. Support during birth giving is supplied by women close by. Having the men present at the birth giving is unfamiliar and unusual [9], and many Somali men do not understand the benefits of being involved in birth giving [8]. There are several stressful moments for Somali women; meeting different people and professionals during pregnancy and birth giving [10], all the equipment and monitoring used in the delivery room, and coming to a hospital for birth giving are all unfamiliar [9]. The birth giving is not so natural as in their home country [5]. Most women in Somalia know that giving birth is something painful, so crying or in other ways showing that you are in pain is shameful. Somali women do not seek help at the hospital if they have pain or are ill. They prefer to be taken care of by friends and family [11]. The family in the Somali culture is so much more than just the family- it is more extended. People nearby, especially women, gather around the woman and gives her attention and support and there is often a trust in God and more seldom a trust in science. A possible consequence is that Somali women refrain from starting the birth giving, since they think that God will decide when it is time to deliver [5].

Many Somali women adopt the approach of “moving on”, they keep their emotions inside themselves and do not wat to share their experiences with people around them [12]. In Sweden there are approximately 38,000 genitally mutilated females, but there are probably higher figures since not everyone is registered or recognised in health care [13]. Many Somali women have been genitally mutilated, which can be a cause of worry. The women are not sure if the HCPs will understand their situation, or if they have knowledge about how to care for a genitally mutilated woman giving birth to a child. The Somali women often experience that they are encouraged to go through a Caesarean section, even though the women themselves want to give birth the natural way [10]. The women felt exposed due to the circumcision; they were presented as teaching examples for students, to demonstrate how a circumcised woman’s genitalia looks [14]. They received hurtful comments about their genital area and experienced a bad approach from HCPs [15], and the women felt that they were doomed to cultural and traditional misunderstandings [14, 16]. In order to create a caring relationship with Somali women there is a need of great engagement from the HCPs and a will to understand and interpret these women’s need.

Relationships are important for these women if they are to feel secure. It has also been shown that more respectful treatment and care is desired by these circumcised women when receiving reproductive health care [16].

There are unsatisfied needs regarding pregnancy and childbirth globally. These are mainly due to language barriers, lack of knowledge about preventive care and complications in relation to childbirth [17]. It has also been shown that in those European countries where the integration policy is insufficient, there is a significantly increased risk for the pregnant woman and the child. So, directed actions and better integration policies towards these immigrant groups will promote health [18]. If the health care should be able to provide good and adequate care and support, there is a need for the HCPs to acquire more knowledge of women from different cultures and their various experiences [17]. The cultural competency is strengthened when caregivers and caretakers show understanding for each other’s life worlds and cultures [19].

It is important to acknowledge Somali women’s lived experiences of pregnancy and birth giving, in order to optimise midwives’ and HCPs’ support during the life-giving process.

A cultural aspect is that Somali women do not want to have dealings with any male HCPs, since it is seen as wrong to be touched by another man outside marriage. So, to be cared for by a male HCP is only accepted if there is a life-threatening situation [5]. This could be experienced as a problem in reproductive health care [16]. Another cultural aspect is that Somali women experience themselves as discriminated against due to their appearance and because they have difficulty making themselves understood [8]. Common language has proven to be important for Somali women in a new society when seeking contact with health care and women’s health care in particular. However, there is scepticism among the Somali women that a third party will be present, for example an interpreter [20]. According to the Somali women, interpreters do not have the competency to use medical terms. Furthermore, the women do not want to share personal issues with an interpreter, no matter whether it is a stranger or someone they know [21]. A meeting should be a meeting between two human beings, which is not achieved in the presence of an interpreter [9]. The Somali women are used to getting information and instruction in a conversation. They are not used to being given information in written form [10]. Good communication affects the encounter with the midwife positively, and it also facilitates understanding and taking part of human rights and responsibilities when Somali women come to a new country. To promote the meeting with Somali woman it has been found that it is important that health care professionals have full focus on the individual woman, as well as use good communication [20].

The aim of the study was to identify and describe Somali women’s lived experience of birth giving in Sweden.

## Method

### Design

To achieve an understanding of Somali women giving birth in Sweden, a qualitative approach with individual interviews and interpretive phenomenological analysis (IPA) was considered most appropriate. Interpretative phenomenological analysis focuses upon people’s understanding of the lived experience, the everyday life and their relationship to the phenomena. The aim was not to generalise but to say something in detail about a specific group experience [22, 23]. The study was conducted in accordance with COREQ (**CO**nsilidated criteria for **RE**porting **Q**ualitative research).

### Setting

A cornerstone in public health work in Sweden is to promote universal access to safe and secure sexuality and good reproductive health thus include also more vulnerable individuals or groups. Maternal health care in Sweden, with its coherent activities and the central role of the midwife, is unique in the world. Maternal health care works with the following areas of activity: health care in connection with pregnancy; support in parenting and parenting groups with childbirth and parental preparation; family planning at the individual level but also gynaecological cell test control for preventing cervical cancer and preventing work regarding living habit.

For health care during pregnancy, there are detailed visitation programs based on current national recommendations within each county. The basic medical program and other care programs are in collaboration with women’s health care. The pregnant woman should be able to come to a midwifery consultation at short notice in case of worrying symptoms or other problems. The midwife makes the first assessment and, if necessary, refers the woman further. If there are medical complications during pregnancy there is obstetrical expertise at the specialist maternal care, which is usually run vy the women’s clinic in the area. The psychosocial or psychological basic program aims to follow the psychological change, support the family’s adaptation to the new situation and identify any need for support.

The Somali women were found in the setting of health care for women in a county in south of Sweden: including a university hospital, and several county hospitals-- all with maternity wards. Some of the Somalian women were working as interpreters and visiting the health care centres as professionals and others were visiting the health care centres as patients.

### Sampling and procedure of data collection

The study adopted purposive sampling as well as snowball sampling to recruit Somali women who had given birth in Sweden. The inclusion criterion was that the women should understand and speak Swedish or English. Using an interpreter was not a choice since it would be a step in the process that could contribute to misinterpretations [24].

The place and the time of the interviews were at the convenience of the women. After the study had been explained to the women, both orally and in writing, they made their own choices regarding participation. Those who consented to be part of the study signed an informed consent. A total of seven women, of fertile age,(20–40 years of age),agreed to participate in the study.

Two women were primiparous and 5 women were multiparous. These women had been residence in Sweden between 5 to 15 years.

An interview guide [23] was developed with two open-ended requests, namely: Please, tell me about your pregnancy and, Please, tell me about your experiences of giving birth in Sweden. To get a deeper understanding and to clarify some parts of the interview, follow-up questions such as ‘Can you tell me more about that?’ or ‘Can you clarify that?’ were asked.

Two test interviews were conducted with those who were invited to participate. These interviews were considered to be of such quality that they were included in the study.

Interviews were conducted in Swedish. Data was collected during autumn 2015 and spring 2016. The interviews lasted from 30 to 80 min (median 55 min) and were digitally recorded with the consent of each participant.

The primary concern of IPA is interpretation of individual experiences. IPA studies benefit from a small number of interviewees [22, 23]. Between three and six interviewees is a reasonable sample size [23].

### Data management and analysis

The verbatim-transcribed interviews were used for the analysis, which was conducted using the IPA [23]. The analysis was performed rigorously, using the six steps of the analytic process. The analysis was performed by two of the researchers. The validity of the findings was increased by the analysis being subjected to peer-debriefing and continuously scrutinised by an additional researcher.

Each interview was analysed separately. To start with, each transcribed interview was read several times so the reader could become familiar with the text. After several readings the interviews were analysed and interpreted using initial noting. Notes were made about aspects of the interviews related to the aim of the study. These initial notes provided the basis for developing different themes. As each transcript was analysed, several noticeable themes were identified and relations between these themes were sought separately for each interview. Noticeable themes were then grouped together from all the interviews, and similar themes were clustered, resulting in four themes. Finally, the themes were interpreted and explained to show similarities and variations as well as experiences [23].

### Trustworthiness/validity

When assessing validity in IPA, the criteria for judging quality by Yardleys [25, 26] are often used. These criteria consider: sensitivity to context, commitment and rigour, transparency and coherence, impact and importance. Sensitivity to context is about preparing oneself for the context by using relevant literature, taking in account ethical issues and how treating empirical data. Commitment and rigour are about methodological competence though data collection and reaching depth and breadth in analysis. Verbatim quotations are included to provide means of validation, showing what was actually said by the interviewees [23].

## Results

The analysis resulted in the identification of four different themes: Being recognised and confirmed as a woman, Communication is important for their independency, Something naturally becomes unknown and complicated, and Professional and competent taking care of. These themes show a unified picture of the Somali women’s lived experience of giving birth in Sweden. The themes varied but were related to each other, had similarities, and showed both positive and negative aspects of the phenomena. The results are described in the text and illustrated with quotations from the interviewees.

### Being recognised and confirmed as a woman

Being pregnant was considered by the Somali woman as something joyful and sacred. The women felt that they had their identity confirmed via their uterus, and being enriched with a child was the meaning of life. The child confirmed the woman, their womanhood.

The women stated that the first impression they had of the midwife was important, that they were approached with a smile, had eye contact and seemed kind. This experience made them feel recognised. When the midwife was sensitive, listening and caring the women felt that there was a personal engagement. The women said that being confirmed as a woman occurred if the midwife showed empathy, consideration and understanding during the pregnancy.

*“I think she was like my mother in some way. She always called me and asked how I.*

*was doing…and she said to me…today we are going to speak English if you want.*

*…I thought that was amazing…she did that for me”* (SW 1).

Physical support such as holding the hand during birth giving and being close was also experienced as worthfully by the women. The women said that there was a deeper relationship and increased confidence between them and the midwife, and that the midwife felt like a close friend or family member.

The Somali women found that the midwives in Sweden had a professional approach. They stated that education that gives knowledge about different cultures is important and this knowledge made the midwives treat all women equally, no matter their background or culture. Women look different, but are treated equally. The women also stated that midwives need empathy and the ability to engage with other people, which is not easy to learn just from literature. When the midwife showed an effort by reading and engage in their culture, the women felt confirmed.

*“ You feel as, you are not left out of it. Everybody is the same…all who are giving.*

*birth, that you are from another country…you don’t experience…you are like all.*

*the others” (SW 7).*

The Somali women said that health care in Somalia functions differently, and that there they would just follow routines and systems, focusing only on the practical issues.

The women stated that in Somalia, pregnancy and birth giving are considered normal and do not require any extra attention or support. So being allowed to show feelings such as crying and being in pain, during both pregnancy and birth giving, and being confirmed in these feelings was valued by the women. This was something that the Somali women were not used to; in their home country it was expected that the women would keep themselves under control. Crying and being sad was seen as a weakness.

The women thought it was important that they had confidence in the midwife and could ask any questions. The Somali women wanted to get clear information and detailed explanations from all health care professionals. The women also felt that it was important that the caring encounter was calm, without stress and with everyone talking calmly. They also experienced themselves as being seen and confirmed if the midwife was patient and took time to listen. Being seen and encouraged by the midwife was experienced by the Somali women as a confirmation that they were managing the situation.

If the women had problems communicating, they experienced being treated differently. If the women thought it was hard to use the Swedish language (and chose not to use an interpreter), the husband accompanied the woman to the visit. So, when information was given the professionals mostly talked to the husband. Sometimes the women had to interrupt and say: *Hey, could you please talk to me or at least look at me, it is my issue.*

Not being recognised due to lack of time or not being present was negatively experienced. One of the Somali women thought it was because she was not Swedish. She did not feel welcome and this made her feel very sad.

The husband’s participation and presence during birth giving were things that the Somali women were not used to. The women stated that this participation was important, since the men then had to experience what the women were going through during birth giving. It helped them understand the pain and realise that the women did not overdo things. The women experienced the support and confirmation given from their husbands as equal to the support the midwife gave.

*“But here (in Sweden) it is different, the men are present, and I think that is.*

*“a big star” that they are present here. They should know what the woman.*

*is going through, what has happened, and he should feel it. That is a good thing,*

*I think”* (SW2).

### Communication is important for their independency

Being dependent on another person was something that the Somali women disliked. Many of the women stated that language plays a central role in helping a person become part of society, making oneself understood and becoming independent.

The Somali women stated that when communicating with health care services it was important to feel they were participating, and this was something they did not experience when an interpreter was used or when the husband was acting as an interpreter. The women found it frustrating not to be able to talk about or explain how they were feeling in their own words. Not being able to communicate independently made them experience being outside. The women found that the interpreter, as well as the husband, only gave a short summary of the situation and problems.

*“My husband was there, interpreting…I didn’t want to…when he was.*

*interpreting it was as if he could say whatever he wanted but not what I.*

*was feeling and I didn’t get the answer I was looking for. The midwife was talking.*

*quite a lot and then she gave me some short information. It felt like…oh no...*

*she said something more. After that, we started to talk in English, and that made.*

*it much easier”* (SW3).

The women stated that the contacts with health care were often private and sensitive conversations, and in these situations they experienced the use of an interpreter negatively.

They were also doubtful about the interpreters being bound by professional secrecy, and so they avoided talking about sensitive issues. It was also stated that they would also recommend other women to use an interpreter. The women strongly disliked being dependent on someone else, like an interpreter. In the communication in health care they instead preferred to use body language, showing and pointing with their fingers and speaking English if possible.

*“I don’t want to have an interpreter, this was private, and this person will not be.*

*bound by professional secrecy. So, I thought I must try…I came to the physician.*

*And I remember that I was telling and showing with my fingers…. ha-ha…You learn”.*

(SW2)

In the encounter with health care the women felt that it was easier to trust and feel confident with a woman. So, they preferred to meet and communicate with female health care professionals.

The women found that in these situations they did not need to pull themselves together or cover themselves. The encounters with male health care professionals were uncomfortable and embarrassing, and the women felt great thankfulness and confidence when meeting female professionals. The Somali women experienced that there was a greater understanding between women. When they met a male professional, they described themselves as more distant and they had difficulty explaining everything. The women felt more independent and not shameful in the intimate situations when there were female health care professionals. They felt independent when they managed to share their feelings with the health care professionals.

To experience trust in an interpretation situation (when interpreters were used) was important, but this was only in the situation with a female interpreter and not a male one.

*“I had interpreters…men…I don’t like that…that men should be interpreters.*

*when I have my visit to the midwife…no I don’t like it…well, about gynae-.*

*cological examinations and embarrassing things…it is not in our culture that’.*

*men should interpret about the genital area”* (SW1).

When, due to the language barrier, the women were captured in dependency, which they disliked, it became a driving force to learn the language. Becoming independent and manage the situation by themselves.

Similar feelings of not participating and not being independent were expressed by the women because of being a stranger in a new country. Not having their family nearby was hard. Not feeling wanted.

and not having somebody to share emotions with about the pregnancy with, made the women feel alone.

*“You are not allowed to work, to go to school. All your friends have the right and.*

*you are alone here. You have no family here, just your husband, no family, no siblings.*

*It is really hard”* (SW6).

After living for a while in Sweden, the women experienced more participation in society, which also affected their experiences of being pregnant and giving birth more positively. Making new friends, learning about their rights, and being independent made them feel joy. In Somalia they were used to having family and friends nearby.

### Something naturally becomes unknown and complicated

Nausea, vomiting and symphysis pubis dysfunction made the Somali women experience the pregnancy as strenuous. These symptoms affected their daily lives and were something they did not expect. There was a conception that the birth giving should be the most troublesome situation, and instead the women perceived the pregnancy to be that. The women could not enjoy their pregnancy and feel happiness about it as they expected. Some of the women had to stay at the hospital, receiving infusions. These experiences during pregnancy were presented as shocking and the situation was out of control. That the HPCs were encouraging and informed them that they were “positively ill”: some symptoms related to the pregnancy-not to a disease, was something that the woman appreciated afterwards. This information made the new phenomenon understandable to them.

*“Yes, the birth giving was supposed to be hard…that’s the picture I had,*

*because if you compare it with pregnancy, I had no idea …I had not thought.*

*about it”* (SW4).

Certain illness conditions, such as diabetes and pre-eclampsia during pregnancy, were unknown to the women. The normal course of pregnancy and birth giving were thereby changed and were experienced as dramatic and strenuous by the women.

Birth giving was also experienced as taking longer than expected; staying at the hospital many hours before the child was delivered caused strain and was strenuous. It was a positive surprise that the birth giving was less painful than expected.

After-pains and bleeding a lot for a long time after birth giving were also unexpected experiences for these women.

The Somali women did not know anything about post-partum depression. In Somalia they do not talk about this condition. Not understanding unknown events that occur during pregnancy and birth giving caused some feelings of frustration and anger.

*“So, I asked her a lot. Especially at the follow-up visit after birth giving, I asked.*

*her about…you can say…I was hit by post-partum depression, you can say….*

*I had no idea”* (SW3).

The women also spoke about complications after birth giving due to genital mutilation. One woman had huge problems with pain and obstipation because of this mutilation. This affected her birth giving experience and she could not feel joy and happiness about having the child. There was also insufficient information about the alleviation of pain during birth giving.

*“I was in pain because of all examinations and starting the birth giving. I think I.*

*could have taken some more? I don’t know…I can’t even recall that.*

*I asked”* (SW5).

*“I have experienced this in both my pregnancies- insufficient information.*

*I told the staff, because I knew that there was help available…”* (SW4).

Events like stillbirths or the child having not expected characteristics were also stated as something unknown and unexpected. One Somali woman was small and thin and gave birth to a baby weighing over four kilos (eight pounds). This was experienced as shocking.

The lived experiences presented here were unknown by the women and they felt that there was a need for more information and support about these different conditions. The women felt fear and worry since they did not know what had hit them. That some of these conditions were normal during pregnancy and birth giving was unknown for them.

The women stated that information about these conditions contributed to a sense of control and a happier pregnancy. Getting information could be crucial for these women regarding what decision to make.

### Professional and competent taking care of

The Somali women found that during the pregnancy and birth giving they met professional and competent HCPs. The women perceived that the professionals focused on them and supplied the care they needed, which contributed to confidence among the women. Regular visits and checks with the midwife during pregnancy were experienced as positive. This accessibility to health care contributed to feeling safety, since the women experienced that the midwife had control over the pregnancy. The midwife also supported the women with some practical issues, which was appreciated because the women in these situations could feel helpless.

That the women with complex illness conditions during the pregnancy and birth giving were examined by gynaecologists and midwives, and that they received treatment, were experienced by the women as benefits which they were grateful for. They were cared for professionally and with competency.

*“I have difficulties in explaining but…you can say that the midwife means a.*

*lot here and plays a big role. They help and play a big role. Well, yes…*

*when I come for checks, I get support”* (SW2).

The women experienced that they were always cared for by well-educated professional and competent health care professionals during pregnancy and birth giving, something they were not used to in Somalia. That the HCPs were bound by professional secrecy and followed rules and laws was described as very important. It was also experienced as secure care, since failures and injuries in the health care were reported. The women said that in Somalia there was not this structure controlling how to manage and act regarding injuries received du to care. In Somalia, nobody knows about mistakes. So, experiences from their home country could create some uncertainty within the women about pregnancy and birth giving in Sweden.

*” …and then the placenta was fast again, and I panicked, and I was bleeding.*

*a lot. I was in panic…I was thinking about Somalia when I had an abrasion without.*

*alleviation of pain. It was terrible”* (SW1).

*“Yes, it was really tough…you can die… all the time I had these thoughts.*

*in my mind. I had heard before that many women die while giving birth.*

*There is no gynaecologist there (in Somalia), and sometimes the women.*

*give birth at home… just women and nobody with experience. In Somalia.*

*there is bombing all the time and physicians are killed* (SW7).

The Somali women expressed apprehension about the HCPs’ knowledge about how genitally mutilated women give birth. Those women who were genitally mutilated shared this insecure feeling and this affected them negatively during both pregnancy and birth giving.

The women experienced discomfort and pain because they had to go through certain procedures in relation to birth giving because of this mutilation. One woman described the strain when the staff had to cut her in the genital area.

*“. then I called my sister and said to her…you will give birth like a princess.*

*How come? she responded…because you have no genital mutilation, I said.*

*You will not have so much pain”* (SW1).

The women pointed out the importance of making HCPs aware about genital mutilation.

The self-image as a genitally mutilated woman who looked different affected the women. As a genitally mutilated woman having caring encounters with HCPs was expressed as hard and embarrassing.

The Somali women talked about feeling insecure regarding the HCPs’ knowledge about women who have been through this genital mutilation. The women could in these caring encounters experience that the staff looked strangely at their genital areas, which made them feel offended. Being treated in this way was described as a very strong experience that affected coming encounters with health care negatively.

*“yes, looking strange, I saw how they looked, and that was hard. I became sad, angry.*

*And I felt that I wanted to call them and tell them that you cannot act like.*

*this, because…I cannot help it, this is something I haven’t chosen. I was so hurt.*

*deep inside and afterwards. You are working it through, understanding”* (SW4).

There was also a feeling of discomfort in the situations when the midwife had to make a gynaecological examination of the women. These examinations could be perceived as painful.

One woman stated that it was strenuous, and it affected her unpleasantly going through these examinations so often.

*“They put fingers in you, assessing how much you have opened. It is terrible.*

*And they are all changing shifts, doing it all the time, I had to tell them, please don’t do it.*

*I was aching down there. My vagina felt like a battlefield* (SW5).

Regarding these women’s lived experiences, they pointed out that it is important that the midwives and the gynaecologists treat them with respect and show understanding about genital mutilation and its problems. Those women with mutilation experienced confidence when the HCPs showed knowledge and understanding about the subject, and supplied professional and competent care. Education and knowledge were the key.

The women also pointed out that they were grateful when they had the possibility to give birth “like all other women”. Coming to a visit before the birth giving and there being reassured that the genital mutilation would not affect the birth giving. This information also contributed to being competently cared for.

*“Yes, being aware that certain people are genitally mutilated. They are.*

*not normal in the genital area…they are different. Having that kind of education.*

*that they should care about everything…”* (SW1).

## Discussion

The findings reflect that health professionals need to have knowledge and understanding regarding cultural aspects when Somali women are giving birth in another context. This study presents the lived experience from the Somali women and their encounters with health care. Acknowledging the cultural aspects may facilitate improvements in reproductive health care and focus on these women’s specific needs. Somali women are a high-risk group that demands special attention and care [27].

Life in the new country, in this case Sweden, differs from life in Somalia. The Somali women feel exposed due to cultural differences. The extended family home at Somalia [5] is not to be found in Sweden, which leads to lower quality of life [28, 29]. The women in Somalia have family and people nearby, especially women, and they gather around the woman and give her attention and support during pregnancy and birth giving [5]. In Sweden, the Somalian women’s network differs from that in Somalia, it is absent or very small and the midwife who has continuous contact with the pregnant Somali woman will become a substitute. The midwife and sometimes HCPs are those who build a trusting relationship with the Somali woman. Due to this relationship, the woman can feel confirmed in the new country, there is a deeper understanding, and sometimes the midwife is seen as a family member. This relationship has been shown to be of importance for the Somali woman. She is acknowledged and confirmed, and if HCPs have a kind approach, smiling and showing understanding, it strengthens these women’s self-image, which is in agreement with other studies [30, 31]. Studies show that when HCPs give time and are personally engaged, the Somali women will perceive themselves as unique and confirmed as women [20, 32].

According to cultural practices, men in Somalia are not involved in childbirth in their home country [33]. In Sweden, the Somali women lacked support from the extended family and they found substitutes in midwives and HCPs, but there was also a new-found support in their husbands. Somali men who had emigrated to the Western World said that they would like to be involved in pregnancy and childbirth [32]. The husband’s participation and presence during birth giving was something new and appreciated by the women. It was considered important as it helped to increase the men’s understanding of what the woman was going through [8].

It was expressed in our study that good communication strengthens the Somali woman’s self-image. Being able to communicate with other people makes them independent. Somali women could experience good communication, even despite poor language ability. Such problems are outweighed by the HCP showing kindness and interest in the person and her background during conversations [30]. The women in our study felt that they were not recognised when HCPs did not communicate directly with them, e.g. when a third party was involved. Inadequate communication is one of the main obstacles to giving care of good quality [34, 35]. Communication may cause a culture clash for these women; they want detailed information but at the same time they do not want to use an interpreter. This is ambiguous, which is also confirmed in another study that Somali women feel suspicion towards interpreters [34], but they can also see the interpreter as a communication resource [30].

However, the individual encounters are important for the Somali women. These encounters should be characterised by respect, kindness and great care, which then strengthen the woman and her experiences of health care encounters as well as society.

A cultural aspect is how pregnancy and birth giving are viewed. It was shown in the present study that there is a need for information about different conditions concerning pregnancy and birth giving, as some symptoms and signs were seen as unknown and complicated by the Somali women. The women expressed fear and uncertainty when they were affected by these symptoms and signs, which included: not being able to manage nausea and vomiting, ending up at the hospital being given infusions, and being convinced that you are going to die when experiencing after-pains following the birth. Post-partum depression was unknown to these women [36]. They said that the word ‘depression’ does not exist in the life world of Somali women and that there is denial about the subject. Mental health problems could be too personal to share [37]. Some problems were not shared by the Somali women with HCPs due to cultural differences between the HCPs and the women. Ignorance of Somali culture and Somali women having unfulfilled expectations created doubt and mistrust towards HCPs [17, 31]. When the HCP showed respect for Somali culture, the women’s experience of care was improved [38]. When HCPs were encouraging and gave verbal information about the natural phenomena related to pregnancy and birth giving the Somali women were relieved [10, 30]. In Somalia, women are supposed to endure pregnancy as well as birth giving. HPCs can strengthen the Somali women when informing them about how we view pregnancy and birth giving in their new country. Giving birth is painful and as a woman you are allowed to show feelings, scream and cry [11, 36].

There is a need for improved and clearer information for these Somali women regarding pregnancy and birth giving. There is also a need for a structure to how this information is given and for adaptation regarding content and format. Group information and video training specifically for Somali women were appreciated by the women as these improved their care satisfaction through providing increased knowledge and reduced stress [39, 40].

Our findings show that genital mutilation affects the Somali women regarding pregnancy and birth giving. During their encounters with health care and HCPs the women realise that they look different in their genital area and HCPs react in different ways, which affects them personally. The women appreciate if they are treated with competency and professionalism like any other woman. They do not want to be discriminated against and want to be informed that they can give birth in a natural way. Some studies confirm that Somali women in the encounter with health care feel differently and that they experience some uncertainty about how they should be treated by HCPs [41, 42].

Due to problems with communication and also emotionally reactions among HCPs there is inadequate care regarding the women who are genitally mutilated [43]. Back home in Somalia genital mutilation is normal; something that is done thoughtfully due to cultural reasons. Being normal and not different is an important issue [44]. Somali women appreciate when there is open communication with HCPs about genital mutilation [41]. There is a need for both knowledge and understanding in order to provide good quality care for these Somali women. Several studies have shown that Somali women mean that there is inadequate treatment, knowledge and understanding for those who are genitally mutilated [30, 32–34, 45, 46]. The issue of genitally mutilation emphasis the importance of being able to establish strategies that improve the quality of medical care for women, and promote a positive childbirth experience, which is in line with the recommendation of WHO [47].

Increased immigration and significant differences in culture between the former country and the new country present us with challenges when it comes to providing good quality care.

### Strengths and limitations

This study used an interpretive phenomenological approach to convey a deeper insight into Somali women’s lived experience of birth giving in Sweden. Purposeful and snowball sampling were used. Snowball sampling may have a limitation that the sample is restricted to a tight circle of acquaintances; however, this was not seen as a problem in our study. Those women who were approached and who declined did so because of scepticism about the recording of the interview, difficulties with the language, and because they did not have time to participate. We are aware that the sample is small but given that Somali women do not like to share their own experiences with others, we had to accept that [4]. Seven women were interviewed, which could be thought of as a small sample. IPA studies benefit from a small number of interviewees, and between three and six interviewees is considered a reasonable sample size [22, 23]. Verbatim quotations are included to show what was actually said by the interviewees, which would enhance the transferability of the findings to a similar group of women.

## Conclusion

The findings show that Somali women consider it important to be confirmed as a woman by the surrounding and professionals during pregnancy and birth giving. The findings in the study indicate that reproductive health care for Somali women should be improved with regard to cultural differences and lived experiences, as these affect their experience of pregnancy and childbirth in Sweden. The midwife and sometimes HCPs are those who build a trusting relationship with the Somali woman. Due to this relationship the woman can feel confirmed in the new country, there is a deeper understanding, and sometimes the midwife is seen as in some way replacing the network they had in their home country. Healthcare personnel who show commitment; a friendly attitude and understanding of the Somali woman’s life and culture, strengthen the women’s self-image and ability to become independent and feel accepted in the new country. There is a need for improved and clearer information for these Somali women regarding pregnancy and birth giving. There is also a need for a structure to how this information is given and for adaptation regarding content and format. Somali women come from a different culture, which affects their lived experiences of pregnancy and birth giving. The women appreciate if they are treated with competency and professionalism; they do not want to be discriminated against. The women feel confidence in health care when they meet competent and professional HCPs. There is a need for both knowledge and understanding in order to provide good quality care for these Somali women, especially those who are genitally mutilated. To increase knowledge and understanding there is a need for future studies, focusing on male participation on prenatal and birth but also exploring health care professionals’ experiences about cultural encounters.,

## Data Availability

The transcripts (in Swedish) from which this manuscript was developed are available on request from the corresponding author.

## Abbreviations

IPA: Interpretative Phenomenological Analysis
HCP: Health Care Professionals
